# The role of recent refugees' educational selectivity in their children's educational decisions in Germany

**DOI:** 10.3389/fsoc.2022.1061976

**Published:** 2023-01-17

**Authors:** Jörg Welker, Gisela Will

**Affiliations:** Leibniz Institute for Educational Trajectories (LIfBi), Bamberg, Germany

**Keywords:** educational selectivity, relative education, first-generation migrants, school placement, educational decisions, adolescents

## Abstract

This paper uses the example of newly arrived refugees to examine the role of recent migrants' educational selectivity in their children's educational decisions in Germany. Building on a theoretical model that understands participation in the educational system as the sum of investment decisions of rational individuals, we assume that positively selected parents are more ambitious about having their children admitted to higher-level secondary schools. The role of parental educational selectivity should be particularly pronounced in federal states in which school administrations allow for greater parental involvement. We use data from the first and second face-to-face interviews of the Refugees in the German Educational System (ReGES) project, with an analytical sample of 1,437 adolescents who came to Germany from Syria, Iraq, Afghanistan, and Iran between 2014 and 2017. To generate a household-level index of educational selectivity, we furthermore rely on various country-of-origin-specific data that we aggregate as reference educational distributions. We run linear probability regression models to analyze the role of parents' educational selectivity in adolescents' school placement. Our findings suggest that parental educational selectivity is beneficial beyond parents' absolute educational levels for adolescents' higher-level school placement. Among the five German federal states represented in our analytical sample, the role of parental selectivity is particularly pronounced in two federal states in which parents are provided with greater possibilities to become involved in their children's educational decisions.

## 1. Introduction

There is increasing academic interest in the role of migrants' educational selectivity in their children's educational success. It is frequently assumed that educational selectivity contributes above and beyond migrant parents' absolute education to the next generation's educational success. While absolute education captures the level of formal instruction that an individual acquired, for example, in the form of educational levels achieved (e.g., ISCED), educational selectivity—or, in other words, relative education—takes into account the country-of-origin-specific value of education and is assumed to proxy latent aspects such as motivation or ability. Indeed, a range of studies suggest that educational selectivity is beneficial for the next generation's educational attainment (e.g., Ichou, 2014; Feliciano and Lanuza, 2017), aspirations (e.g., Engzell, 2019), and educational decisions (e.g., Brunori et al., 2020; Tong and Harris, 2021). These studies focus primarily on migrant children who were born in the receiving country and are therefore second-generation migrants. We aim to contribute to quantitative research by analyzing the link between parents' educational selectivity and the educational participation of children who, like their parents, are themselves first-generation migrants.

We do so by using the example of newly arrived refugees^1^ in Germany. The arrival of many families with school-aged children in the course of recent refugee immigration gives us the opportunity to examine the integration of a sufficiently large group of first-generation migrant students who are admitted to the German school system as lateral entrants. We understand integration as the inclusion of individuals into the social systems of a society and focus on the dimension of structural integration in this paper. Structural integration describes the placement of individuals in the institutional and economic systems of a society (Esser, 2000)—in our case, the placement of migrant adolescents in educational institutions in Germany. Although we refer to a sample of refugees, we believe that our findings generally apply to first-generation migrants. Previous research found that mechanisms discussed in the context of social and ethnic educational inequality also help explain the educational success of refugees (e.g., Will and Homuth, 2020; Schipolowski et al., 2021), even if some individual preconditions or opportunity structures may be systematically different for refugees.

We focus on a highly relevant educational decision—enrollment in higher-level secondary school—and aim to answer the following research question: What role does immigrant parents' educational selectivity play for first-generation migrant adolescents' school placement in Germany? Successful integration into the educational system is vital because it not only determines their educational outcomes, such as the degrees that they will likely complete, but also affects their chances in later life. In Germany, migrants of school age are assigned to school types immediately after their arrival or after a relatively short time in the place of destination. In this respect, school placement can be seen as an early integration outcome. Because the German educational system is strictly organized according to tracks, assignments to a particular school type significantly influence the subsequent educational trajectories of young students, not least because subsequent changes of school tracks are extremely difficult.

We assume that family background, as indicated by parents' educational selectivity, can to some extent help explain the school placement of migrant children who enter the educational system laterally. To our knowledge, the role of immigrant selectivity has thus far not been taken into account to explain the integration of first-generation migrants into the secondary level of school, neither for refugees nor other migrant groups. We assume that the mechanisms by which selectivity contributes to explaining the educational decisions of immigrant children are identical for refugees and other first-generation immigrants. We will explicitly point out when we expect differences that relate to the specific situation of refugees.

The paper is structured as follows: section Overview of the research gives a brief overview of the current state of the research on the role of educational selectivity, with a particular focus on consequences for migrant children's educational outcomes. Theoretical considerations are presented in section Educational selectivity and adolescent educational decisions. We model integration into the educational system as rational choice decisions and link this model to mechanisms that are expected to explain the role of educational selectivity in migrants' societal integration. Section Research design describes the research design and the data. To analyze the role of parents' educational selectivity for children's placement in higher-level secondary school, we run linear probability regression models. Descriptive and multivariate findings are presented and discussed in section Results.

## 2. Overview of the research

It is well established that parental education is essential for children's education (e.g., Erikson and Jonsson, 1996). This finding also holds for migrants (e.g., Brinbaum and Cebolla-Boado, 2007; Kristen and Granato, 2007) and, more particularly, the group of refugees who migrated to Germany in recent years (Will and Homuth, 2020; Schipolowski et al., 2021). Most of the previous studies share an understanding of parents' educational background in terms of absolute degrees. However, the value of education is highly context dependent, particularly among migrants. Consider two individuals who obtained their highest educational qualifications in two different countries. Although these two qualifications may be equivalent to the same (absolute) educational level—for example, higher education—their relative value is conditioned by the position that they convey to their holders in the respective reference population. While higher education is common in many countries, it may be attained only by a minority in other countries, thus making it relatively more valuable in the latter case because the share of less educated individuals in the population is greater there.

In studies that include migrants from different origin countries, such differences are overlooked if only absolute levels of education are accounted for. In the context of international migration, it is particularly relevant to evaluate an individual's educational attainment relative to the context in which it was achieved because migrants are usually selected on education, with the better educated often being more likely to migrate (Feliciano, 2005b; Spörlein et al., 2020). This finding is essential as a descriptive contribution, and it is consequential for migrants' integration into the host society in various dimensions.

Among the consequences of educational selectivity, outcomes that refer to the next generation's education may be the best empirically confirmed. Previous research analyzed the role of educational selectivity for the second generation's educational attainment (Feliciano, 2006b, 2018; Ichou, 2014; Feliciano and Lanuza, 2017; van de Werfhorst and Heath, 2019) as well as for a range of educational outcomes that influence children's later educational attainment, such as expectations and aspirations (Feliciano, 2006a,b; Engzell, 2019; Cebolla-Boado et al., 2021; Nygård, 2021; Tong and Harris, 2021) and educational decisions (Feliciano, 2005a, 2006b; van de Werfhorst et al., 2014; Engzell, 2019; van de Werfhorst and Heath, 2019; Brunori et al., 2020; Tong and Harris, 2021).

Overall, these studies point to a positive contribution of educational selectivity to their children's education. Among the studies that specifically deal with consequences for educational decisions, findings suggest that educational selectivity is positively associated with second-generation migrant adolescents' chances of attending an academic secondary track (van de Werfhorst et al., 2014; Engzell, 2019; van de Werfhorst and Heath, 2019). For the United States, educational selectivity is found to increase migrant children's chances of college enrollment (Feliciano, 2005a, 2006b; Tong and Harris, 2021). As a special case of educational decisions, Brunori et al. (2020) analyze the role of parents' educational selectivity in their children's school dropout and find that positive educational selectivity decreases the likelihood of an early dropout.

Some of these studies measure selectivity on the group level, comparing the educational levels of a migrant group—for instance, Turks in Germany—to those of the origin population (e.g., Feliciano, 2005a, 2006a; van de Werfhorst and Heath, 2019), while others investigate the role of individual-level (i.e., parental) educational selectivity (e.g., Ichou, 2014; Engzell, 2019). Our paper applies the latter approach because it accounts for variation within migrant groups, which is essential for our research interest in investigating the consequences of parental selectivity. Despite using different measures, most cited studies share assumptions about mechanisms that might explain positive consequences of educational selectivity for the next generation's education. Three mechanisms are frequently considered to play a role: motivation, relative status maintenance, and skills.

First, parental educational selectivity could play a role in children's educational outcomes because selectivity is supposed to be a proxy for unobserved motivational attributes. According to this reasoning, better educated individuals should be more ambitious (Chiswick, 1999). In this context, motivation is frequently used as an umbrella term under which researchers subsume aspects such as drive for success or achievement orientation (e.g., Ichou, 2014; Feliciano and Lanuza, 2017). Positively selected parents might have greater ambitions for their children's education and pass motivational attributes down to their children so that their children have greater ambitions themselves.

Second, educational selectivity reflects an individual's position in the educational distribution of the origin society and can be seen as an indicator of the social status that migrants hold prior to migration. Positively selected migrants are assumed to have occupied a higher social rank in the place of origin. This premigration status may also be relevant in the place of destination because much of migrants' behavior may be guided by the position they hold in their societies of origin (Feliciano, 2005a; Ichou, 2014). This expectation is particularly important in the context of the next generation's educational success in the place of destination because education is an essential means to maintain a family's social status across generations.

Third, some researchers see educational selectivity as an indicator of cognitive skills (Ichou, 2014; Spörlein and Kristen, 2019a). These could result in greater resources, such as cultural or social capital (Spörlein and Kristen, 2019a), which might give positively selected migrants better access to relevant information and helpful strategies to support their children. Skill advantages could also be transmitted to their children (Schulz et al., 2017) so that the children of positively selected parents should themselves have greater abilities for success.

While the previously mentioned studies analyze the role of educational selectivity for educational outcomes among students who mostly spent all or most of their childhood in the destination country, we focus on refugee adolescents as a group of first-generation migrants who immigrated to Germany at school age. The mechanisms that drive the intergenerational consequences of educational selectivity should also apply to first-generation migrants. However, certain conditions under which they participate in the educational system in the place of destination are specific for this group. Most importantly, they entered the German educational system laterally instead of starting their school career in the place of destination.

## 3. Educational selectivity and adolescent educational decisions

### 3.1. Modeling migrant families' educational behavior

In the following subsection, we link the mechanisms that we expect to drive the consequences of educational selectivity to a general model that explains educational behavior. This model understands integration into the educational system as the sum of investment decisions of rational individuals. Expected costs and benefits and the probabilities of realizing different options determine individuals' educational behavior (Erikson and Jonsson, 1996; Breen and Goldthorpe, 1997; Esser, 1999). Educational outcomes are shaped by individual motivation, resources, and institutional opportunities and restrictions (Diehl et al., 2016). In our study, adolescents are the central individuals of interest, but context persons—most importantly parents, who influence their children's educational behavior and partially make educational decisions for them—can be equally relevant actors.

Families can anticipate a range of benefits from investments in their children's education. Education is a precondition for success later in life (Erikson and Jonsson, 1996; Breen and Goldthorpe, 1997). Families with greater cultural resources may be particularly aware of the benefits of education (Bourdieu and Passeron, 1990). Positively selected families are expected to possess greater cultural resources and attribute greater value to education.

However, investments in education generate costs (Erikson and Jonsson, 1996; Breen and Goldthorpe, 1997). Higher-level secondary education usually lasts longer, and it is more uncertain whether children will successfully complete this path compared to shorter and less demanding educational trajectories. Parents with a greater propensity to delay gratification may be more likely to opt for more demanding school types for their children (Erikson and Jonsson, 1996). Educational selectivity is frequently seen as a proxy for such motivational resources (Spörlein and Kristen, 2019a); therefore, positively selected families may be more willing to have their children admitted to higher-level secondary school.

Further nonmonetary costs can arise from the desire for status maintenance, which posits that parents and children want to avoid downward intergenerational mobility (Breen and Goldthorpe, 1997). For recent migrants, the social status they have in the place of origin is assumed to be more relevant than their current status in the place of arrival (Ichou, 2014). Families may want to avoid downward assimilation and want their children to attain an educational level that allows them to have a comparable or even higher social status than their parents had in the place of origin. To match their parents' premigration status, children of migrants who are better educated based on the standards of the origin country are expected to have to invest more in education than children of migrants who are relatively less educated.

The status attainment assumption can furthermore be linked to the assumption that migrants who are positively selected on education should also be positively selected on motivational attributes: Higher-status families might have higher educational aspirations than lower-status families (Erikson and Jonsson, 1996). Aspirations may be seen as an expression of motivational characteristics through which parental educational selectivity might reflect on children's educational decisions. Migrant parents with higher premigration status might also be more motivated to make investments, for instance, in destination-language acquisition, which should ultimately foster their children's educational success in the destination country. Additionally, parents' motivational attributes are resources that may, at least partially, be transmitted to their children and influence their children's behavior. Accordingly, children of positively selected parents might themselves have higher aspirations, be willing to invest more, and make more ambitious educational decisions (Ichou, 2014).

Based on these theoretical considerations, we propose our first hypothesis:

H1: Children from positively selected households have a higher likelihood of higher-level secondary school placement.

### 3.2. Migrant-specific conditions in the German educational system

Specific structural conditions for newcomers could moderate how parents' educational selectivity is reflected in their children's educational success. School-aged immigrants generally have the right and the obligation to attend school in Germany, but regulations at the federal state level specify the organization of lateral entrants' integration into the school system (Massumi et al., 2015). In many federal states, new immigrants are usually initially taught in separate classes for new immigrants. While these classes in some federal states (e.g., Bavaria and Saxony) are especially established at lower-level school types, regulations in other federal states attribute more weight to previous educational experiences and individual achievements when newcomers are assigned to a school type (see Will and Homuth, 2020).

This consideration of individual experiences and achievements is usually accompanied by greater opportunities for parents to exert influence. For instance, regulations in Rhineland-Palatinate explicitly point out the responsibilities of parents (Ministerium für Bildung., 2017). Parental involvement is also referenced in regulations in North Rhine-Westphalia and Hamburg (Behörde für Schule und Berufsbildung Hamburg., 2018; Ministerium für Schule und Bildung des Landes Nordrhein-Westfalen., 2018). In such contexts, positively selected parents could be more persistent in trying to have their children admitted to higher-level school types. We therefore derive another hypothesis with regard to the school placement of newly arrived immigrants who enter the educational system laterally, focusing on the potentially more important role of educational selectivity in some federal states:

H2: The role of parental educational selectivity in higher-level school placement is particularly pronounced in federal states that allow for greater involvement of parents in their children's school placement.

## 4. Research design

### 4.1. Destination-specific data source

Our analyses rely on data from the Refugees in the German Educational System (ReGES) project, which provides longitudinal data on the educational trajectories of young refugees who came to Germany between 2014 and 2017. Children and adolescents living with at least one parent were sampled using a multistage design. First, five of the 16 federal states in Germany were selected: Bavaria, Hamburg, North Rhine-Westphalia, Rhineland Palatinate, and Saxony. These five states vary according to various macrolevel indicators relevant for the integration of immigrants, such as unemployment rates or experience with immigrant integration, as well as in their way of integrating newly arrived immigrants into schools. Based on the general population registers of the municipalities that were selected within these five federal states, target persons who fulfilled the criteria (e.g., age, nationality, and date of arrival) were sampled (for further details, see Steinhauer et al., 2019).

The adolescent participants were surveyed a total of seven times between 2018 and 2020 at intervals of 5 months on average (for further information, see Will et al., 2021). At the time of the sampling, they were 14–16 years old and were assumed to be at the end of the first stage of secondary schooling. To obtain richer contextual information on the adolescents' family background, their parents were also interviewed at the first measurement time on various topics, such as their highest educational degree in the place of origin. If the parents did not want to take part, the adolescents themselves were asked some questions about their family backgrounds. Our analyses focus on data from two waves. Most variables—particularly those related to family background—were measured at the first face-to-face interview. The second face-to-face interview, conducted approximately 1 year after the first face-to-face interview, is the source of a range of variables that refer to the destination context, including our outcome variables.

Our analytical sample consists of adolescents who completed valid interviews in the first and second face-to-face waves and for whose parents we have the necessary information on educational background. Because measuring educational selectivity depends on the availability of origin-specific datasets (see subsection Operationalizing educational selectivity), our sample is restricted to the four largest groups in the ReGES study: Syrians, Iraqis, Afghans, and Iranians. We additionally exclude students who did not transition into regular classes. In Germany, recent migrants are often enrolled in special newcomer classes. Such classes may be set up in various types of schools. Attendance of newcomer classes at a certain type of school can influence the type of school that a student will attend after transferring to a regular class but by no means determine the later school type. Thus, analyzing the educational placement of students who did not enter regular classes would be associated with many uncertainties. We further exclude one case for which information about the attended school type is missing. This procedure results in an analytical sample of 1,437 adolescents in 1,310 families.

### 4.2. Operationalizing educational selectivity

Measuring educational selectivity first and foremost requires information on the educational degrees that parents acquired in their place of origin. In the ReGES project, the respondents were asked for their highest country-of-origin-specific educational qualification. Subsequently, each qualification was coded according to the internationally comparable ISCED97 classification (see UNESCO Institute for Statistics, 2012).

The information on absolute educational levels is then used to calculate an index of educational selectivity, following the relative education approach (Ichou, 2014). The central objective of this approach is to determine a migrant's position in the educational distribution of a certain reference population. In our study, the reference population is equivalent to the population of the origin country where refugee parents grew up and acquired their education.

To generate reference educational distributions for the origin groups that we consider, four large-scale datasets serve as sources. The datasets for Syria, Iraq, and Afghanistan were collected under the Multiple Indicator Cluster Surveys (MICS) program (Central Bureau of Statistics et al., 2008; Central Statistics Organisation, and UNICEF, 2013; Central Statistical Organization, 2019). We rely on the data from the most recent survey years for each origin country, which are 2006 for Syria, 2010/2011 for Afghanistan, and 2018 for Iraq. The Iranian data are a two percent public use sample of the 2011 National Population and Housing Census (Minnesota Population Center, 2020).

Before aggregating the microdata into educational distributions, we dropped all observations without valid information on educational attainment. Because average educational levels may systematically differ between men and women, older and younger generations, and wealthier and poorer regions within an origin country, we generate educational distributions that are specific to gender, five-year age group, and subnational region of origin.^2^ For this reason, cases with missing values for gender, age, or region are also excluded. With these restrictions, the origin-specific data sources account for 54,525 individuals in Afghanistan, 66,851 in Syria, 78,493 in Iraq, and 1,115,084 in Iran.^3^

Based on the respective origin-specific educational distribution, the relative education of a ReGES respondent is calculated by adding the shares of the reference population with lower educational levels plus half of the reference population with the same educational level as the respondent.^4^ All values of relative education range on a continuum between 0 and 1. For instance, a value of 0.6 indicates that an individual is at least as educated as 60 percent of the origin population of the same gender, age group and subnational region of origin. Individuals with a relative education value above 0.5 are better educated than half of the reference population and therefore characterized as positively selected, whereas values below this threshold indicate negative selectivity.

We expand the measurement of relative education in one essential aspect. Looking at individual-level relative education would be of limited informative value for the analyses of intergenerational processes, where it is not sufficient to consider the background of only one parent. We therefore operationalize educational selectivity as the highest relative education in the household, that is, the highest relative education of the responding parent or his or her partner.

In addition to the measurement of relative education, our multivariate models include the highest educational level completed in the household (HISCED) as a measurement of parents' absolute education prior to migration. The HISCED variable is recoded and includes the following categories: primary school or below (HISCED 0-1), lower secondary school (HISCED 2), upper secondary (HISCED 3) and postsecondary education (HISCED 4-6).

### 4.3. Further explanatory and outcome variables

The multivariate models contain further variables that could generally explain variation in migrants' school placement. Control variables include adolescents' age in years (at the time of sampling), gender, legal status (insecure vs. secure), number of months since they arrived in Germany, and extent to which they have a place to retreat. Additionally, the potential experience of traumatizing events before or during migration could hamper students' educational integration (Qureshi et al., 2011). We account for this by including an indicator of PTSD risk, measured with a scale assessing ten symptoms. We recoded the scale score into a binary variable: respondents who reported three or fewer symptoms are considered to be at low risk, whereas those who reported four or more symptoms are considered to have at least a medium PTSD risk. For further details on the scale, see Boillat and Chamouton (2013). In addition, we control for whether the parents participated in the first interview.

Because we expect state-level differences in students' school placement, dummy variables represent the five federal states in which the ReGES study was conducted. To examine our second hypothesis, which posits that the role of parental educational selectivity in higher-level school placement is particularly pronounced in federal states that allow for greater parental involvement, we include interactions for the federal states and the highest relative education in the household in the multivariate models referring to school placement. While in Bavaria and Saxony, schooling for new immigrants is initially primarily provided in less-demanding school types, parents are expected to play a more important role in school type assignment in Hamburg, North Rhine-Westphalia and Rhineland-Palatinate.

In addition, we include some variables that could be associated with both the outcome variable and parental educational selectivity: adolescents' educational aspirations, their German language skills, and their school performance in the place of origin. Educational aspirations are often related to educational outcomes (e.g., Dollmann, 2017) and may be expressions of motivational attributes, which are supposedly captured in the measure of relative education (Ichou, 2014). We therefore consider adolescents' idealistic aspirations. These are included as a dummy variable that distinguishes between aspirations to acquire a qualification that allows one to attend university vs. aspirations for lower degrees. German language skills have been found to be acquired more quickly by adolescents from positively selected refugee families (Welker). Similarly, these should be positively associated with the likelihood of attending higher-level secondary schools because greater skills may increase chances of entering more demanding school types (e.g., Stanat and Edele, 2016). However, it should be emphasized that language skills are not just a prerequisite of schooling. Causality may also run in the opposite direction: Attending higher-level secondary school may result in better German skills if students enrolled in more demanding school contexts have a steeper learning curve. Thus, we believe that it is important to include a measure of German skills in our analyses, but at the same time, we will refrain from interpreting potential effects causally because better language skills might also be a consequence of being enrolled in higher-level secondary school. In the ReGES study, German language competency tests were conducted to assess adolescents' destination-language skills. We rely on sum scores of the Peabody Picture Vocabulary Test (PPVT; Lenhard et al., 2015), which assessed the adolescents' receptive German vocabulary at the time of the first interview. As an indicator of adolescents' educational experience, we consider their school performance in the place of origin, which was reported by the parents or self-assessed by those adolescents whose parents did not participate in the survey. The performance assessments range on a scale from 0 to 100 and are centered on the country-of-origin mean for the multivariate analyses. This variable should be associated not only with adolescents' school placement in Germany but also with parents' relative education, as we expect students' origin-specific performance to reflect their parents' relative education. This may be related to motivational attributes, social status prior to migration, or transmitted cognitive skills.

To obtain information on our outcome variable, that is, school-type placement, adolescents were asked about their educational episodes in Germany. Secondary schools in Germany can be summarized as follows: higher-level secondary schools grant students a degree of direct access to university education, whereas intermediate-level and lower-level secondary schools usually prepare students for vocational training. In addition, there are school types that join more than one track, such as combined schools for lower-level and intermediate-level tracks and comprehensive schools that integrate all three tracks. Given the age range of the ReGES sample, schooling was no longer mandatory for some adolescents at the time of the second face-to-face interview. For these students, undergoing vocational training or vocational preparation are alternatives to transitioning into upper secondary school. We operationalize school placement as a dummy variable: students at higher-level secondary schools vs. all other adolescents, including those who had already left the general school system.

For a descriptive overview of all variables, see Table 1. Missing values are multiply imputed (m = 25) using predictive mean matching on all independent variables except our central independent variable, that is, parental educational selectivity, and all variables necessary to generate the index of educational selectivity (i.e., parents' absolute education, gender, age, origin country, and origin region). All missing values resulted from item nonresponse, which we expect to be missing at random. We estimate linear probability models with robust standard errors for both outcomes.

**Table 1:** Descriptive sample characteristics (*N* = 1,437).

**Variables**	**N/n**	**Freq/mean**	**SD**	**Range**
Higher-level secondary school placement				
Higher-level secondary school	206	14.34		
Other school type or out of school	1,231	85.66		
Total	1,437	100.00		
Highest relative education in household	1,437	0.71	0.24	0.04; 1.00
Highest absolute education in household				
HISCED 0/1	558	38.83		
HISCED 2	188	13.08		
HISCED 3	284	19.76		
HISCED 4/5/6	407	28.32		
Total	1,437	100.00		
Gender of adolescent				
Female	643	44.75		
Male	794	55.25		
Total	1,437	100.00		
Age of adolescent				
14 years	576	40.08		
15 years	488	33.96		
16 years	373	25.96		
Total	1,437	100.00		
Months since immigration to Germany	1,437	29.86	8.84	3; 53
Legal status of adolescent				
Insecure	378	27.17		
Secure	1,013	72.83		
Total	1,391	100.00		
PTSD risk				
Low risk	1,133	87.56		
Medium or high risk	161	12.44		
Total				
Place to retreat				
No	206	14.50		
Rarely	186	13.09		
Sometimes	445	31.32		
Most of the time	584	41.10		
Total	1,421	100.00		
Parental participation in wave 1				
No	364	25.33		
Yes	1,073	74.67		
Total	1,437	100.00		
Federal state				
Bavaria	161	11.20		
Hamburg	103	7.17		
North Rhine-Westphalia	885	61.59		
Rhineland-Palatinate	194	13.50		
Saxony	94	6.54		
Total	1,437	100.00		
School performance at origin (centered on country mean)	1,427	0.00	26.13	−75.85; 38.80
Educational aspirations of adolescent				
Qualification that allows to attend university	1,075	75.23		
Other	354	24.77		
Total	1,429	100.00		
German language skills (PPVT sum score)	906	92.02	31.50	3; 206

Values displayed in this table are based on nonimputed data. Source: ReGES parent data (wave 1) and adolescent data (waves 1 and 4).

## 5. Results

### 5.1. Descriptive analyses

**Figure 1:**
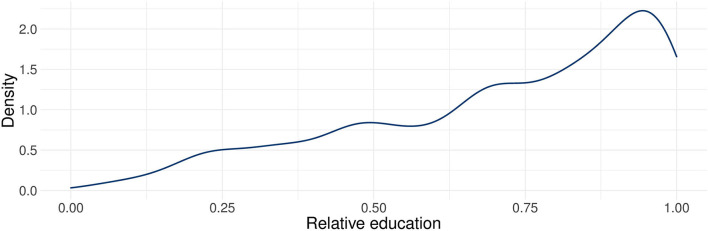
Density distribution of the highest relative education in the household (*N* = 1,437). Source: ReGES parent and adolescent data (wave 1).

To gain an overview of our central explanatory variable, Figure 1 displays the density distribution of the ReGES household highest relative education. The distribution is skewed to the left, representing the high shares of refugees who are positively selected on education. The median relative education of this analytical sample is 0.78, which expresses that the relatively best educated parent of the median household is at least as educated as 78 percent of the population of the place of origin. Despite its skewness, the distribution covers the whole spectrum from negative to positive selectivity.

Examining the relation between relative and absolute education, we find that both measures are strongly correlated (Spearman's rho = 0.86). In some respects, this is a limitation to exploring the respective roles of both measures in the multivariate analyses, but we nonetheless believe that it is important to include both for several reasons. Most importantly, educational degrees, which are measured by absolute levels, could have some kind of signaling effect (Bol and van de Werfhorst, 2011) that is not reflected in the measure of relative education, whereas latent aspects such as motivational resources should be better captured by the measure of relative education. In addition, the value of (absolute) educational level strongly differs between migrants from different origin countries. This may best be illustrated by an example: According to the origin-specific data that we use to generate the relative education index, 19.3% of the Syrian population completed secondary or higher levels of education, while comparable levels of education were completed by only 8.7% of the Afghan population (Central Bureau of Statistics et al., 2008; Central Statistics Organisation, and UNICEF, 2013). Therefore, having at least a secondary degree is of greater value in Afghanistan than in Syria. This origin-specific value of education is only considered in the measure of relative education.

**Figure 2:**
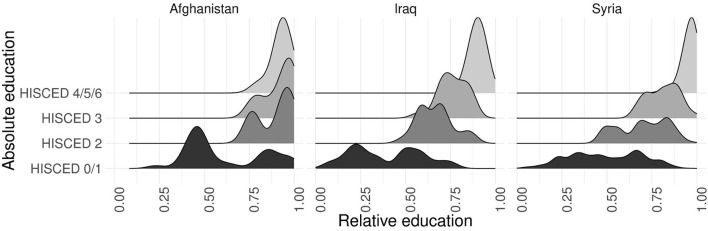
Density distributions of the highest relative education in the household by HISCED level and origin group (Afghanistan: *n* = 89; Iraq: *n* = 167; Syria: *n* = 1,157). Because of the few cases from Iran (*n* = 24), density distributions are not displayed for this origin group. Source: ReGES parent and adolescent data (wave 1).

Figure 2 depicts the density distributions of relative education by HISCED level and origin group (except for households from Iran, which are too few cases to be presented separately). Particularly among lower levels of absolute education, the households cover a wide range of the relative education index and strongly overlap with other HISCED categories. This applies to all three origin groups displayed in the figure but is most striking in the case of Afghans, among whom even low absolute levels such as primary education can result in highly positive educational selectivity. There are important differences in regard to households where at least one parent completed lower secondary education (HISCED 2): While among Afghan households, a lower secondary degree already results in extremely positive educational selectivity, the picture is more nuanced among Iraqi and Syrian households, where lower secondary degrees cover a wider range on the selectivity index. However, the graph also shows that among all three origin groups, households where at least one parent completed postsecondary or higher education are concentrated in an extreme peak at the upper end of the relative education scale. This suggests that the latter are a highly select group within the sample.

We now turn to the description of our outcome variable. Students at higher-level secondary schools make up 14.3% of the relevant analytical sample. Other students were mostly enrolled in intermediate-level schools (19.6%), followed by comprehensive (16.7%), lower-level (16.2%), and combined secondary school types (12.6%). A small share of 4.5% were enrolled in other, not further specified schools. The remainder of the sample (16.1%) had left the general educational system and were mostly receiving vocational training or vocational preparation at the time of the second interview. Differentiating by adolescents' school type, the median household has a relative education index of 0.90 among students at higher-level secondary schools. In contrast, the median relative education index is 0.73 for households of students who did not attend higher-level secondary school. This gap of 17% points suggests that children from positively selected families have greater chances of attending higher-level schools.

### 5.2. Multivariate analyses

In the following subsection, we present the findings of our multivariate analyses of the role of parental educational selectivity for young refugees' school placement (Table 2). The first model includes the measure of parental relative education and controls (Model 1). Relative education is positively associated with adolescents' chances of attending higher-level secondary school. Adolescents from families where at least one parent is at the top of the relative education distribution have a 24.8% greater chance of attending higher-level secondary school than (hypothetical) adolescents whose parents are at the bottom of the relative education index. Because the first model does not include the measure of absolute education, the size and strength of the association between relative education and school placement are likely overestimated. Turning to the covariates included in this model, we especially see that macrolevel factors have some importance as rather strong differences exist between federal states. Compared to adolescents in Bavaria, their counterparts in three other federal states—Hamburg, Rhineland-Palatinate, and North Rhine-Westphalia—have substantially greater chances of being enrolled in higher-level secondary schools. The latter federal states are the same for which we expect a greater role of parental selectivity. We will get back to this finding in more detail in Model 5, where we additionally consider interactions between federal states and parental selectivity. Among the other controls, gender and PTSD risk appear to play some role, with males and adolescents with a medium or high PTSD risk being less likely to attend higher-level schools.

**Table 2:** Linear probability models of adolescents' higher-level secondary school placement.

	**Model 1**	**Model 2**	**Model 3**	**Model 4**	**Model 5**
Highest relative education in household	0.248^***^		0.123^*^	0.101	−0.047
	[0.036]		[0.056]	[0.055]	[0.070]
Highest absolute education in household (ref: HISCED 0/1)					
HISCED 2		−0.008	−0.034	−0.042	−0.042
		[0.022]	[0.025]	[0.025]	[0.025]
HISCED 3		0.081^**^	0.041	0.027	0.025
		[0.025]	[0.032]	[0.031]	[0.031]
HISCED 4/5/6		0.153^***^	0.096^**^	0.066	0.062
		[0.024]	[0.036]	[0.036]	[0.036]
Gender of adolescent (ref: female)	−0.049^**^	−0.046^*^	−0.046^*^	−0.039^*^	−0.039^*^
	[0.018]	[0.018]	[0.018]	[0.018]	[0.018]
Age of adolescent	0.013	0.011	0.012	0.013	0.013
	[0.011]	[0.011]	[0.011]	[0.011]	[0.011]
Duration of stay in Germany in months	−0.002	−0.001	−0.001	−0.002	−0.002
	[0.001]	[0.001]	[0.001]	[0.001]	[0.001]
Legal status of adolescent (ref: insecure)	−0.032	−0.034	−0.035	−0.038	−0.038
	[0.022]	[0.022]	[0.022]	[0.022]	[0.022]
PTSD risk	−0.052^*^	−0.051^*^	−0.050^*^	−0.036	−0.037
	[0.024]	[0.024]	[0.024]	[0.024]	[0.024]
Place to retreat	0.000	−0.001	−0.000	−0.002	−0.003
	[0.009]	[0.009]	[0.009]	[0.009]	[0.009]
Parental interview completed	−0.009	−0.019	−0.017	−0.021	−0.021
	[0.022]	[0.022]	[0.022]	[0.022]	[0.022]
Federal state (ref: Bavaria)					
Hamburg	0.175^***^	0.173^***^	0.173^***^	0.156^***^	0.131
	[0.042]	[0.042]	[0.042]	[0.043]	[0.113]
Rhineland-Palatinate	0.100^***^	0.108^***^	0.104^***^	0.103^***^	−0.083
	[0.030]	[0.030]	[0.030]	[0.030]	[0.084]
North Rhine-Westphalia	0.112^***^	0.108^***^	0.108^***^	0.096^***^	−0.022
	[0.022]	[0.021]	[0.021]	[0.021]	[0.045]
Saxony	0.056	0.065	0.060	0.053	−0.118
	[0.034]	[0.034]	[0.034]	[0.034]	[0.083]
School performance at origin				0.001^**^	0.001^**^
				[0.000]	[0.000]
Educational aspirations of adolescent				0.069^***^	0.070^***^
				[0.017]	[0.017]
German skills of adolescent (PPVT)				0.001^*^	0.001^*^
				[0.000]	[0.000]
Interaction: relative education ^*^ Hamburg					0.029
					[0.173]
Interaction: relative education ^*^ Rhineland-Palatinate					0.260^*^
					[0.128]
Interaction: relative education ^*^ North Rhine-Westphalia					0.171^*^
					[0.075]
Interaction: Relative education ^*^ Saxony					0.244
					[0.141]
Constant	−0.207	−0.075	−0.134	−0.230	−0.131
	[0.176]	[0.176]	[0.177]	[0.179]	[0.182]
Observations	1,437	1,437	1,437	1,437	1,437
Adjusted R2	0.0490	0.0548	0.0568	0.0796	0.0797

Robust standard errors in brackets.

^*^p < 0.05, ^**^p < 0.01, ^***^p < 0.001.

Source: ReGES parent data (wave 1) and adolescent data (waves 1 and 4).

Before running a model that includes both relative and absolute parental education, we take a closer look at the role of absolute education (Model 2). As expected, there is a positive relationship between absolute parental education and the outcome. This association can be found for adolescents whose parents attained upper secondary education—these have an 8.1% greater chance of attending higher-level secondary school—and is even stronger for children whose parents attained postsecondary or higher education and who have a 15.3% greater chance of higher-level secondary school placement, compared to adolescents whose parents completed at most primary education. Besides the measure of absolute education, this model contains the same covariates as the previous model. The strength and size of these covariates' association with the outcome are almost identical in both models.

As we include both relative and absolute parental education in one model (Model 3), the association between relative education and adolescents' school placement is indeed reduced but remains significant. Keeping absolute parental education constant, adolescents from a perfectly positively selected household still have a 12.3% greater chance of attending higher-level secondary school compared to adolescents from a perfectly negatively selected household. At the same time, the parents' absolute educational levels matter: children whose parents attained postsecondary or higher education have 9.6% greater chances of attending a higher-level school. However, in contrast to the previous model, parental upper secondary education is no longer significantly associated with the outcome, and the association between higher than secondary parental education and the outcome is weaker. We assume that both in regard to relative and absolute education, parts of the diminished associations are caused by the strong correlation between both measures. These findings nevertheless suggest that both absolute and relative parental education play a role in adolescents' educational decisions, which supports our first hypothesis: educational selectivity is beneficial for first-generation migrants' enrollment in higher-level secondary schools, over and above the contribution of the parents' absolute educational levels. However, we also acknowledge that the model explains only a rather small share of variation (Adj. R2 = 0.0568) in these adolescents' higher-level secondary school attendance. Regarding the covariates, their associations with the outcome variable are comparable in strength and size to the previous models.

Model 4 additionally considers the adolescents' educational aspirations, their German skills, and their school performances at origin, which might potentially be drivers of the association between educational selectivity and adolescents' school placement. All three variables are positively associated with placement in higher-level secondary schools. Compared to the previous model, the variation explained by this model increases slightly by—after all—more than two percentage points, which underlines the role of aspirations, language skills, and previous school performances in adolescents' school-type attendance. The importance of previous school performances is in line with regulations in many federal states that stipulate previous educational experience as a basis for the decision of which school type an adolescent is admitted to. We assumed that educational aspirations, language skills, and school performance at origin may be associated not only with the outcome but also with educational selectivity: children from positively selected families may have greater aspirations, which translates into a greater likelihood of being enrolled in higher-level secondary school. Additionally, they may have performed better at school in their place of origin and have advantages in acquiring German language skills. This may explain why parental relative education no longer plays a significant role in this model and why the size of its association with the outcome is further reduced compared to Model 3. However, the reduction of the association with the outcome applies not only to relative education but also to absolute education, as parental postsecondary or higher education is no longer significantly associated with adolescents' school placement. As far as our controls are concerned, PTSD risk is no longer significantly associated with the outcome.

To examine our second hypothesis, which posits that the role of parental educational selectivity in higher-level school placement is particularly pronounced in federal states that provide more freedom of choice, we consider interactions of parental selectivity and the federal states in Model 5. As mentioned above, the previous models show that students in some federal states have significantly greater chances of attending higher-level schools. Compared to Bavaria, this applies to all other federal states in the sample except Saxony. By including interactions, we aim to analyze these association in more depth. These results reveal a significant contribution of selectivity in two states. In North Rhine-Westphalia and Rhineland-Palatinate, greater chances of attending higher-level secondary school, which we saw in the previous models, are significantly associated with educational selectivity. In other words, first-generation migrant children from positively selected families in these two federal states have a greater likelihood of being enrolled in higher-level secondary schools than their counterparts in Bavaria, for whom we supposed that schooling regulations provide only a few opportunities for parental involvement. In North Rhine-Westphalia and in Rhineland-Palatinate, students from positively selected households have a substantially greater chance of attending higher-level secondary schools. This finding supports hypothesis H2 for two of the three federal states for which we expected a more pronounced role of parental selectivity. In contrast, we cannot confirm our hypothesis for Hamburg, for which we also expected greater possibilities of parental involvement. The greater chances of Hamburg students attending higher-level secondary school, which we see in the previous models, do not appear to be driven by educational selectivity but by other factors. Regarding our covariates, we still see an effect of gender, with males having substantially lesser chances to attend higher-level schools. School performances at origin, educational aspirations and German skills are farther positively associated with the adolescents' school placement in Germany. However, this full model still explains only a rather small share of variation (Adj. R2 = 0.0797), which we acknowledge to be a limitation to our analyses.

## 6. Conclusion

In this paper, we focused on the intergenerational role of migrants' educational selectivity, more precisely in regard to their descendants' educational participation in Germany. Using a sample of young refugees who entered the German educational system laterally and who are themselves first-generation migrants, we analyzed whether and to what extent their parents' relative education is reflected in their school placement. We assumed that their parents' educational selectivity could be beneficial for these adolescents' educational decisions. The data that we used to answer our research questions were particularly suited to test our hypotheses. The ReGES data not only contain information on the school placement of a large number of newly immigrated adolescents but also detailed information on the parents, which makes it possible to create a comparatively differentiated index of relative education.

Our findings lend some support to our hypotheses on young immigrants' school placement: we see a positive relationship between relative education and attendance of a higher-level secondary school in the bivariate analyses and the multivariate models, which only loses its significance when we additionally control for mechanisms that might contribute to explaining this relationship. This is in line with previous research, which shows a similar role of parental educational selectivity for the educational success of 1.5- or second-generation migrants. Although first-generation migrants face specific conditions that accompany their integration into the educational system in Germany, the intergenerational transmission of education also plays an essential role in these young migrants' educational success.

We also see that the role of parental selectivity is particularly pronounced in North Rhine-Westphalia and Rhineland-Palatinate. We assumed that this is because regulations in these federal states provide students and their parents with more freedom of choice in the school placement decision and therefore enable the mechanisms assumed to be related to relative education to unfold better. However, our analyses show no comparable findings for Hamburg, although we assumed that families could also have more leeway in assigning newly arrived immigrants to a type of school there. Analyses that build on our findings and take a closer look at school enrollment processes in North Rhine-Westphalia and Rhineland-Palatinate might provide clues as to how the relative educational background of parents may be taken into account in a supportive manner in this context. However, it becomes clear that mechanisms of educational selectivity can hardly be effective if there is no leeway for young people and parents when making educational decisions. This is all the more regrettable because the resources, such as unobserved cognitive and socioemotional skills, associated with parents' educational selectivity can positively contribute to their descendants' educational success and their integration into the educational system. In order to utilize these resources, it would be advisable to provide families with opportunities of involvement in their children's educational decisions. The federal states should review their schooling regulations and consider giving families more say and, thus, the possibility to unfold the potential of positively selected migrants.

Our findings also show that parents' absolute educational levels are positively associated with their children's school placement in Germany. Overall, our results attest to the general importance of parental educational background—both absolute and relative—in this matter. The highly relevant role of family background has important implications, considering the fact that being assigned to a particular type of secondary school strongly predetermines students' subsequent educational trajectories. Integrating newcomer students into a highly stratified educational system, such as in Germany, makes it all the more important to support immigrant parents in enrolling their adolescent descendants. If better educated families are more likely to have their children enrolled in higher-level schools, additional support might especially be necessary for less educated families, in which children benefit less from the intergenerational transmission of advantages. Additional support should be given in order to ensure that recent migrant children have equal chances regardless of their family background.

Parents are usually the most important figures for underage students and are therefore normally involved in all decisions about their educational careers. However, migrants who only arrived recently in their place of destination may not always possess all the necessary information to make such important decisions. Therefore, it is important to support newly arrived immigrants and their parents—irrespective of their educational background—to help them make informed decisions. Furthermore, while it is to be assessed positively that migrants of school age have the right and obligation to attend school soon after their arrival in Germany, early decisions about the school type that newcomers attend should not be irreversible. The permeability of different school tracks plays an important role here and is perhaps even more important for newly arrived students than for students who have spent their entire school career in the host country.

A limitation of our findings on higher-level secondary school placement may be stated in regard to the low level of variation that our multivariate models explain. In addition to parents' relative and absolute education, other factors for which we do not control appear to be decisive for young migrants' school placement. Some of this variation is likely explained by structural conditions. We assumed that school-type decisions of recent refugees who entered the German educational system laterally are only to some extent genuinely taken by these refugee adolescents and their parents. Their scope of decision-making takes place within a framework of regulations and is influenced by decisions made by school authorities (Will et al., 2022). In addition, it can be assumed that influences at the municipal level (e.g., support potential, existing schools) also play an important role. In our paper, we specifically focused on intergenerational aspects of educational selectivity on educational decisions, barely scratching the surface of the importance of structural conditions for this matter.

Finally, while our findings suggest some evidence for a significant role of educational selectivity on early integration outcomes that goes beyond the role of absolute parental educational levels, it is also conceivable that the benefits of positive selectivity come into play more strongly with a longer duration of stay. For instance, a longer duration of stay in the place of destination has been found to be relevant in explaining better labor market outcomes of positively selected migrants (Schmidt et al., 2021). Future research should therefore also examine the potential consequences of parents' educational selectivity on the medium- and long-term integration of newcomer students into the educational system and the labor market at the destination.

## Data availability statement

The original contributions presented in the study are included in the article/supplementary material, further inquiries can be directed to the corresponding author.

## Ethics statement

Ethical review and approval was not required for the study on human participants in accordance with the local legislation and institutional requirements. Written informed consent to participate in this study was provided by the participants' legal guardian/next of kin.

## Author contributions

All authors listed have made a substantial, direct, and intellectual contribution to the work and approved it for publication.

## Footnotes

^1^This paper uses the term “refugees” as a collective term for all individuals seeking humanitarian protection. The notion is not limited to the strict definition of the Geneva Convention.

^2^We rely on a region-of-origin-specific measurement of relative education, which has been found to measure educational selectivity more accurately than a country-of-origin measurement if migrants' origins concentrate on few subnational regions of a country and if wealth is unequally distributed between subnational regions of a country (see Spörlein and Kristen, 2019b). Both aspects apply to the origin groups considered in this paper.

^3^These large case numbers allow us to divide the data into gender-, age-, and region-specific groups that mostly are sufficiently large to serve as reference for the generation of the relative education index. In few cases, for which the size of the reference group is below 50 individuals, we ran plausibility checks by comparing the region-specific selectivity index to an index that is not region specific. Because the latter is only specific to age groups and gender for each origin country, it consists of larger reference groups. The plausibility checks revealed no substantial deviations between both indices among these cases, so we consistently use the region-specific index for all cases.

^4^Because we generate a relative education index that is specific to gender, age group, and origin region, this information is required not only for the origin population but also for the ReGES parents. Adolescents whose parents did not participate in the ReGES study were not asked about their parents' age. To avoid excluding these cases, their mothers' and fathers' age is approximated by the average age of the mothers or fathers for whom this information is available, who come from the same origin country, and whose children have the same age.
